# Complete Genome Sequences of *Lactobacillus johnsonii* Strain N6.2 and *Lactobacillus reuteri* Strain TD1

**DOI:** 10.1128/genomeA.00397-14

**Published:** 2014-05-08

**Authors:** Michael T. Leonard, Ricardo B. Valladares, Alexandria Ardissone, Claudio F. Gonzalez, Graciela L. Lorca, Eric W. Triplett

**Affiliations:** Department of Microbiology & Cell Science, University of Florida, Gainesville, Florida, USA

## Abstract

We report here the complete genome sequences of *Lactobacillus johnsonii* strain N6.2, a homofermentative lactic acid intestinal bacterium, and *Lactobacillus reuteri* strain TD1, a heterofermentative lactic acid intestinal bacterium, both isolated from a type 1 diabetes-resistant rat model.

## GENOME ANNOUNCEMENT

Several lactic acid bacteria, including *Lactobacillus johnsonii* and *Lactobacillus reuteri*, were identified in the type 1 diabetes-resistant rat model (BioBreeding diabetes-prone rats [BB-DR]) (1). While *L. johnsonii* N6.2 feeding was associated with a reduction of diabetes prevalence in BioBreeding diabetes-prone (BB–DP) rats (2), *L. reuteri* strain TD1 exhibited a similar onset of type 1 diabetes as that of the untreated rats (2). Additionally, *L. reuteri* is known to secrete the antimicrobial compound reuterin (3); however, strain TD1 does not possess the genes required for reuterin production. As part of an effort to further investigate the roles of *L. johnsonii* N6.2 and *L. reuteri* TD1 in diabetes development, the complete genome sequences of each strain are reported here.

Both *L. johnsonii* strain N6.2 and *L. reuteri* strain TD1 were isolated from the stools of BB-DR rats (2), and their genomes were sequenced at the University of Florida Interdisciplinary Center for Biotechnology Research (UF-ICBR) using the PacBio SMRT system (Pacific Biosciences, Menlo Park, CA, USA). From N6.2, a total of 38,960 reads were obtained, with a mean read length of 3,110 bp. From TD1, a total of 86,930 reads were obtained, with a mean read length of 5,217 bp. Additionally, N6.2 was sequenced using two lanes of the Illumina GAII platform, yielding 203 million reads at 10,740× coverage (Illumina, Inc., San Diego, CA). An initial set of 29 contigs was obtained for N6.2 using the CLC Genomics Workbench and CLC finishing module (CLC, Inc., Aarhus, Denmark). A set of 7 contigs was obtained for TD1 when assembled using Celera Assembler version 7.0 software (4). A single scaffold was obtained for both assemblies by detecting overlaps with Mauve 2.3.1 (5) and manually assembling the remaining contigs. Both initial genome assemblies were further refined using the PacBio RS_Resequencing.1 module with Quiver consensus calling. The final circular genomes of N6.2 and TD1 have 1,887,251 bp and 2,145,445 bp, respectively, with overall G+C contents of 34.5% and 38.8%, respectively. The scaffolds were subject to an NheI restriction digest (*in silico*) and verified against an OpGen optical map of N6.2 and TD1 using the same enzyme (6). Open reading frame (ORF) prediction and annotation were performed through the Rapid Annotations using Subsystems Technology (RAST) pipeline (7) and verified using Glimmer (8), RNAmmer (9), and tRNAscan-SE (10).

By these analyses, 1,728 and 1,962 protein-coding ORFs were detected in the circular chromosomes of N6.2 and TD1, respectively. Fifty-five tRNAs and 4 rRNA operons, composed of 5S, 16S, and 23S rRNA genes, were detected in the genome of N6.2. Seventy tRNAs and 6 rRNA operons were detected in TD1.

### Nucleotide sequence accession numbers.

The results of this whole-genome shotgun project have been deposited with GenBank under accession no. CP006811 and CP006603.
